# 3-[2-(Anilinocarbon­yl)eth­yl]-1-methyl-1*H*-imidazolium hexa­fluorido­phosphate

**DOI:** 10.1107/S1600536808005291

**Published:** 2008-03-20

**Authors:** Wen Pei, Xiaoyang Si, Dengxiang Ji, Jianbing Ji

**Affiliations:** a College of Chemical Engineering and Materials Science, Zhejiang University of Technology, Hangzhou 310014, People’s Republic of China

## Abstract

The title compound, C_13_H_16_N_3_O^+^·PF_6_^−^, which has an imide group in the imidazolium cation, is a new ionic liquid above its melting point. Two neighbouring mol­ecules are connected by a weak non-classical C—H⋯O hydrogen bond with the formation of centrosymmetric 14-membered dimers.

## Related literature

For the preparation of the compound, see: Yang *et al.* (2007 ▶).
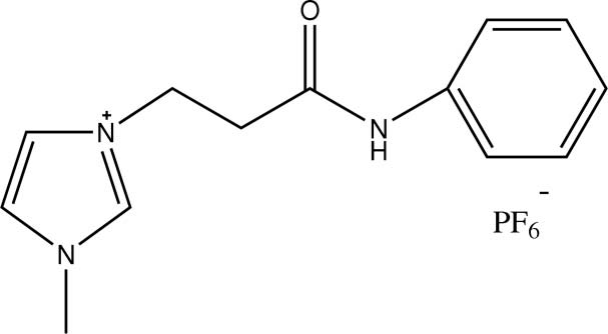


## Experimental

### 

#### Crystal data

C_13_H_16_N_3_O^+^·PF_6_^−^
*M* *_r_* = 375.26
Monoclinic, 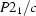

*a* = 9.6414 (4) Å
*b* = 19.4934 (10) Å
*c* = 8.8402 (4) Å
β = 103.6880 (11)°
*V* = 1614.27 (13) Å^3^
*Z* = 4
Mo *K*α radiation
μ = 0.24 mm^−1^
*T* = 296 (1) K
0.40 × 0.30 × 0.27 mm

#### Data collection

Rigaku R-AXIS RAPID diffractometer
Absorption correction: multi-scan (*ABSCOR*; Higashi, 1995 ▶) *T*_min_ = 0.879, *T*_max_ = 0.937
3677 measured reflections
3677 independent reflections
2362 reflections with *F* ^2^ > 2σ(*F* ^2^)
*R*_int_ = 0.037

#### Refinement

*R*[*F* ^2^ > 2σ(*F* ^2^)] = 0.056
*wR*(*F* ^2^) = 0.145
*S* = 1.01
3677 reflections
218 parameters
H-atom parameters constrained
Δρ_max_ = 0.50 e Å^−3^
Δρ_min_ = −0.38 e Å^−3^

### 

Data collection: *PROCESS-AUTO* (Rigaku, 1998 ▶); cell refinement: *PROCESS-AUTO*; data reduction: *CrystalStructure* (Rigaku/MSC, 2004 ▶); Larson (1970 ▶); program(s) used to solve structure: *SIR92* (Altomare *et al.*, 1994 ▶); program(s) used to refine structure: *CRYSTALS* (Betteridge *et al.*, 2003 ▶); molecular graphics: *ORTEP-3* (Farrugia, 1997 ▶); software used to prepare material for publication: *CrystalStructure*.

## Supplementary Material

Crystal structure: contains datablocks global, I. DOI: 10.1107/S1600536808005291/rk2076sup1.cif

Structure factors: contains datablocks I. DOI: 10.1107/S1600536808005291/rk2076Isup2.hkl

Additional supplementary materials:  crystallographic information; 3D view; checkCIF report

## Figures and Tables

**Table 1:** Hydrogen-bond geometry (Å, °)

*D*—H⋯*A*	*D*—H	H⋯*A*	*D*⋯*A*	*D*—H⋯*A*
C12—H12⋯O1^i^	0.93	2.23	3.119 (3)	160

Symmetry code: (i) 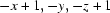.

**Fig. 1.**
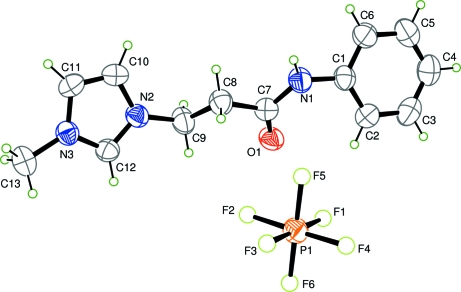
View of the title molecule with the atom-labelling scheme. Displacement ellipsoids are drawn at the 50% probability level. H atoms are presented as a small spheres of arbitrary radius.

**Fig. 2.**
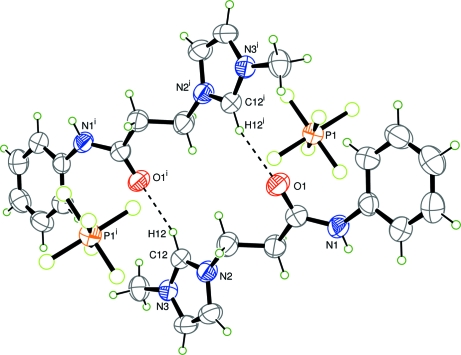
View of a hydrogen-bonded (dashed lines) dimers with the symmetry code: (i) 1 - x, -y, 1 - z.

## supplementary crystallographic information

### Comment

The phenol and aniline are two vital infectant in water. We prepared the new ionic liquid with a imide group in the imidazolium cations to extracted and seperate phenol and aniline from the water and achieved good result.

Two neighbour molecules are connected by a weak, non-classical, C12–H12···O1^i^ H-bonds with the formation centrosymmetrical 14-membered dimers (symmetry code: (i) 1 - *x*, -*y*, 1 - *z*).

### Experimental

The title compound was prepared according to the procedure of Yang *et al.* (2007). A mixture of 3-chloro-*N*-phenylpropanamide (9.18 g, 0.05 mol) and 1-methylimidazole (4.1 g, 0.05 mmol) in 30 ml of acetonitrile was heated with stirring at 353 K for 9 h. The solvent was removed by distillation, and a gray liquid was obtained. Then 50 ml of water and KPF_6_ (10.15 g, 0.055 mol) was added, the mixture was stirred at ambient temperature for 24 h. On completion, the water were filtered off and the title compound was obtained as a white solid in 61% yield. Diffraction quality crystals were obtained by slow evaporation of an ethylacetate solution at room temperature.

### Refinement

All H atoms were placed in calculated posistion for N–H = 0.86 Å, C–H = 0.97Å (for CH_2_), C–H = 0.96Å (for CH_3_), C–H = 0.93Å (for aromatic) and included in the final cycles of refinement as riding mode with *U*_iso_(H) = 1.2*U*_eq_(parent atom).

### Crystal data

**Table:**

C_13_H_16_N_3_O^+^·P_1_F_6_^–^	*F*_000_ = 768.00
*M**_r_* = 375.26	*D*_x_ = 1.544 Mg m^−^^3^
Monoclinic, *P*2_1_/*c*	Mo *K*α radiation λ = 0.71075 Å
Hall symbol: -P 2ybc	Cell parameters from 9621 reflections
*a* = 9.6414 (4) Å	θ = 3.0–27.4º
*b* = 19.4934 (10) Å	µ = 0.24 mm^−^^1^
*c* = 8.8402 (4) Å	*T* = 296 (1) K
β = 103.6880 (11)º	Block, colourless
*V* = 1614.27 (13) Å^3^	0.40 × 0.30 × 0.27 mm
*Z* = 4	

### Data collection

**Table:**

Rigaku R-AXIS RAPID diffractometer	2362 reflections with *F*^2^ > 2σ(*F*^2^)
Detector resolution: 10.00 pixels mm^-1^	*R*_int_ = 0.037
ω–scan	θ_max_ = 27.4º
Absorption correction: multi-scan(ABSCOR; Higashi, 1995)	*h* = −12→12
*T*_min_ = 0.879, *T*_max_ = 0.937	*k* = −25→0
3677 measured reflections	*l* = 0→11
3677 independent reflections	

### Refinement

**Table:**

Refinement on *F*^2^	*w* = 1/[0.0013*F*_o_^2^ + 5.0000σ(*F*_o_^2^)]/(4*F*_o_^2^)
*R*[*F*^2^ > 2σ(*F*^2^)] = 0.056	(Δ/σ)_max_ < 0.001
*wR*(*F*^2^) = 0.145	Δρ_max_ = 0.50 e Å^−^^3^
*S* = 1.01	Δρ_min_ = −0.38 e Å^−^^3^
3677 reflections	Extinction correction: Larson (1970)
218 parameters	Extinction coefficient: 192 (26)
H-atom parameters constrained	

### Special details

**Table:**

Refinement. Refinement using all reflections. The weighted *R*-factor (*wR*) and goodness of fit (*S*) are based on *F*^2^. *R*-factor (gt) are based on *F*. The threshold expression of *F*^2^ > 2.0σ(*F*^2^) is used only for calculating *R*-factor (gt).

### Fractional atomic coordinates and isotropic or equivalent isotropic displacement parameters (Å^2^)

**Table:**

	*x*	*y*	*z*	*U*_iso_*/*U*_eq_	
P1	0.66835 (6)	0.13474 (4)	0.87558 (8)	0.0527 (2)	
F1	0.80918 (19)	0.10215 (11)	0.8443 (2)	0.0979 (7)	
F2	0.57794 (19)	0.09494 (9)	0.7276 (2)	0.0832 (5)	
F3	0.5293 (2)	0.16754 (12)	0.9074 (2)	0.1199 (8)	
F4	0.7630 (2)	0.17460 (10)	1.0203 (2)	0.0966 (6)	
F5	0.6809 (2)	0.19681 (9)	0.7656 (2)	0.0946 (6)	
F6	0.6574 (2)	0.07272 (11)	0.9846 (2)	0.1142 (8)	
O1	0.78438 (18)	0.06145 (9)	0.4653 (2)	0.0562 (5)	
N1	0.8257 (2)	0.17224 (11)	0.4113 (2)	0.0540 (6)	
N2	0.3528 (2)	0.07578 (10)	0.2310 (2)	0.0486 (5)	
N3	0.1253 (2)	0.06435 (10)	0.1961 (2)	0.0461 (5)	
C1	0.9762 (2)	0.17472 (12)	0.4617 (2)	0.0477 (6)	
C2	1.0511 (2)	0.13719 (13)	0.5867 (3)	0.0604 (8)	
C3	1.1984 (3)	0.14078 (16)	0.6293 (3)	0.0685 (9)	
C4	1.2713 (3)	0.18264 (16)	0.5499 (3)	0.0659 (9)	
C5	1.1969 (3)	0.22013 (14)	0.4270 (3)	0.0671 (9)	
C6	1.0501 (2)	0.21689 (12)	0.3810 (3)	0.0589 (8)	
C7	0.7400 (2)	0.11745 (12)	0.4140 (2)	0.0467 (7)	
C8	0.5835 (2)	0.13090 (12)	0.3475 (3)	0.0536 (7)	
C9	0.5065 (2)	0.06525 (12)	0.2934 (3)	0.0587 (8)	
C10	0.2893 (2)	0.11482 (13)	0.1046 (2)	0.0606 (8)	
C11	0.1480 (2)	0.10802 (14)	0.0839 (2)	0.0596 (8)	
C12	0.2500 (2)	0.04552 (12)	0.2837 (2)	0.0469 (6)	
C13	−0.0136 (2)	0.03930 (13)	0.2138 (3)	0.0607 (8)	
H1	0.7839	0.2098	0.3749	0.065*	
H2	1.0024	0.1094	0.6424	0.072*	
H3	1.2486	0.1147	0.7124	0.082*	
H4	1.3703	0.1854	0.5795	0.079*	
H5	1.2462	0.2485	0.3732	0.081*	
H6	1.0010	0.2427	0.2967	0.071*	
H10	0.3359	0.1412	0.0442	0.073*	
H11	0.0783	0.1291	0.0073	0.071*	
H12	0.2638	0.0159	0.3685	0.056*	
H81	0.5726	0.1621	0.2600	0.064*	
H82	0.5427	0.1515	0.4270	0.064*	
H91	0.5201	0.0338	0.3808	0.070*	
H92	0.5466	0.0454	0.2126	0.070*	
H131	−0.0048	0.0238	0.3187	0.073*	
H132	−0.0446	0.0020	0.1430	0.073*	
H133	−0.0823	0.0758	0.1912	0.073*	

### Atomic displacement parameters (Å^2^)

**Table:**

	*U*^11^	*U*^22^	*U*^33^	*U*^12^	*U*^13^	*U*^23^
P1	0.0535 (4)	0.0500 (4)	0.0539 (4)	−0.0018 (3)	0.0113 (3)	0.0002 (3)
F1	0.0664 (11)	0.1231 (17)	0.1046 (14)	0.0181 (11)	0.0211 (10)	−0.0216 (12)
F2	0.0852 (12)	0.0635 (11)	0.0860 (11)	−0.0082 (8)	−0.0097 (9)	−0.0124 (9)
F3	0.0853 (14)	0.162 (2)	0.1203 (17)	0.0382 (14)	0.0409 (13)	−0.0086 (15)
F4	0.1166 (15)	0.0890 (14)	0.0679 (11)	0.0010 (11)	−0.0106 (10)	−0.0213 (10)
F5	0.1389 (17)	0.0636 (11)	0.0753 (11)	−0.0261 (11)	0.0136 (11)	0.0114 (9)
F6	0.1306 (18)	0.1012 (16)	0.1067 (15)	−0.0211 (13)	0.0199 (13)	0.0490 (12)
O1	0.0532 (10)	0.0470 (10)	0.0682 (11)	0.0004 (8)	0.0141 (8)	0.0127 (8)
N1	0.0485 (11)	0.0400 (12)	0.0690 (14)	0.0004 (9)	0.0050 (10)	0.0066 (10)
N2	0.0452 (11)	0.0438 (11)	0.0566 (12)	−0.0013 (9)	0.0114 (9)	0.0057 (9)
N3	0.0449 (11)	0.0423 (11)	0.0513 (11)	−0.0017 (9)	0.0115 (9)	−0.0019 (9)
C1	0.0485 (13)	0.0406 (13)	0.0524 (14)	−0.0029 (11)	0.0086 (11)	−0.0069 (11)
C2	0.0622 (17)	0.0636 (17)	0.0514 (15)	−0.0057 (13)	0.0059 (13)	0.0049 (13)
C3	0.0626 (18)	0.073 (2)	0.0603 (17)	0.0050 (15)	−0.0033 (14)	−0.0012 (15)
C4	0.0497 (15)	0.076 (2)	0.0702 (18)	0.0004 (14)	0.0102 (15)	−0.0162 (16)
C5	0.0625 (18)	0.0656 (19)	0.0763 (19)	−0.0155 (14)	0.0227 (16)	−0.0096 (16)
C6	0.0641 (17)	0.0466 (15)	0.0630 (16)	−0.0047 (12)	0.0093 (14)	0.0008 (13)
C7	0.0483 (14)	0.0455 (14)	0.0477 (14)	0.0031 (11)	0.0141 (11)	−0.0002 (11)
C8	0.0479 (14)	0.0491 (15)	0.0623 (16)	0.0013 (11)	0.0099 (12)	0.0012 (12)
C9	0.0437 (13)	0.0504 (15)	0.0793 (19)	−0.0014 (11)	0.0091 (12)	0.0053 (13)
C10	0.0631 (17)	0.0627 (17)	0.0556 (15)	−0.0049 (13)	0.0133 (13)	0.0175 (13)
C11	0.0589 (17)	0.0611 (17)	0.0529 (15)	0.0034 (13)	0.0016 (13)	0.0161 (13)
C12	0.0503 (14)	0.0405 (13)	0.0489 (13)	0.0002 (10)	0.0100 (11)	0.0024 (10)
C13	0.0470 (14)	0.0621 (17)	0.0745 (18)	−0.0067 (12)	0.0172 (13)	−0.0082 (14)

### Geometric parameters (Å, °)

**Table:**

P1—F1	1.581 (2)	C5—C6	1.378 (3)
P1—F2	1.5920 (18)	C7—C8	1.508 (3)
P1—F3	1.569 (2)	C8—C9	1.500 (3)
P1—F4	1.5878 (18)	C10—C11	1.337 (4)
P1—F5	1.5745 (19)	N1—H1	0.860
P1—F6	1.565 (2)	C2—H2	0.930
O1—C7	1.220 (2)	C3—H3	0.930
N1—C1	1.414 (3)	C4—H4	0.930
N1—C7	1.354 (3)	C5—H5	0.930
N2—C9	1.469 (3)	C6—H6	0.930
N2—C10	1.371 (3)	C8—H81	0.970
N2—C12	1.329 (3)	C8—H82	0.970
N3—C11	1.363 (3)	C9—H91	0.970
N3—C12	1.319 (2)	C9—H92	0.970
N3—C13	1.468 (3)	C10—H10	0.930
C1—C2	1.378 (3)	C11—H11	0.930
C1—C6	1.391 (3)	C12—H12	0.930
C2—C3	1.383 (4)	C13—H131	0.960
C3—C4	1.373 (4)	C13—H132	0.960
C4—C5	1.363 (3)	C13—H133	0.960
			
F1—P1—F2	89.22 (10)	N3—C11—C10	107.2 (2)
F1—P1—F3	179.54 (12)	N2—C12—N3	108.8 (2)
F1—P1—F4	89.07 (10)	C1—N1—H1	116.3
F1—P1—F5	90.36 (11)	C7—N1—H1	116.4
F1—P1—F6	89.02 (12)	C1—C2—H2	120.0
F2—P1—F3	91.21 (10)	C3—C2—H2	120.0
F2—P1—F4	178.00 (12)	C2—C3—H3	119.8
F2—P1—F5	88.52 (9)	C4—C3—H3	119.8
F2—P1—F6	91.50 (10)	C3—C4—H4	120.3
F3—P1—F4	90.50 (11)	C5—C4—H4	120.3
F3—P1—F5	89.49 (12)	C4—C5—H5	119.3
F3—P1—F6	91.13 (13)	C6—C5—H5	119.3
F4—P1—F5	90.45 (10)	C1—C6—H6	120.3
F4—P1—F6	89.51 (10)	C5—C6—H6	120.3
F5—P1—F6	179.38 (13)	C7—C8—H81	109.2
C1—N1—C7	127.3 (2)	C7—C8—H82	109.2
C9—N2—C10	126.9 (2)	C9—C8—H81	109.2
C9—N2—C12	125.2 (2)	C9—C8—H82	109.2
C10—N2—C12	107.8 (2)	H81—C8—H82	109.5
C11—N3—C12	108.7 (2)	N2—C9—H91	108.8
C11—N3—C13	126.36 (19)	N2—C9—H92	108.8
C12—N3—C13	124.9 (2)	C8—C9—H91	108.8
N1—C1—C2	122.6 (2)	C8—C9—H92	108.8
N1—C1—C6	118.1 (2)	H91—C9—H92	109.5
C2—C1—C6	119.3 (2)	N2—C10—H10	126.3
C1—C2—C3	120.1 (2)	C11—C10—H10	126.3
C2—C3—C4	120.5 (2)	N3—C11—H11	126.4
C3—C4—C5	119.3 (2)	C10—C11—H11	126.4
C4—C5—C6	121.4 (2)	N2—C12—H12	125.6
C1—C6—C5	119.4 (2)	N3—C12—H12	125.6
O1—C7—N1	123.4 (2)	N3—C13—H131	109.5
O1—C7—C8	122.1 (2)	N3—C13—H132	109.5
N1—C7—C8	114.5 (2)	N3—C13—H133	109.5
C7—C8—C9	110.5 (2)	H131—C13—H132	109.5
N2—C9—C8	112.3 (2)	H131—C13—H133	109.5
N2—C10—C11	107.5 (2)	H132—C13—H133	109.5
			
C1—N1—C7—O1	1.5 (4)	C13—N3—C12—N2	177.3 (2)
C1—N1—C7—C8	−178.3 (2)	N1—C1—C2—C3	179.0 (2)
C7—N1—C1—C2	−34.4 (4)	N1—C1—C6—C5	−179.7 (2)
C7—N1—C1—C6	145.6 (2)	C2—C1—C6—C5	0.3 (3)
C9—N2—C10—C11	177.3 (2)	C6—C1—C2—C3	−1.0 (4)
C10—N2—C9—C8	61.7 (3)	C1—C2—C3—C4	1.3 (4)
C9—N2—C12—N3	−177.1 (2)	C2—C3—C4—C5	−0.8 (4)
C12—N2—C9—C8	−122.1 (2)	C3—C4—C5—C6	−0.0 (4)
C10—N2—C12—N3	−0.3 (2)	C4—C5—C6—C1	0.2 (4)
C12—N2—C10—C11	0.7 (2)	O1—C7—C8—C9	−23.5 (3)
C11—N3—C12—N2	−0.1 (2)	N1—C7—C8—C9	156.3 (2)
C12—N3—C11—C10	0.5 (3)	C7—C8—C9—N2	178.7 (2)
C13—N3—C11—C10	−176.8 (2)	N2—C10—C11—N3	−0.7 (3)

### Hydrogen-bond geometry (Å, °)

**Table:**

*D*—H···*A*	*D*—H	H···*A*	*D*···*A*	*D*—H···*A*
C12—H12···O1^i^	0.93	2.23	3.119 (3)	160

Symmetry codes: (i) −*x*+1, −*y*, −*z*+1.
